# Mid-Cavitary Obstruction in Hypertrophic Cardiomyopathy (HCM): A Rare Case Report and Management Approach

**DOI:** 10.15388/Amed.2025.32.1.16

**Published:** 2025-02-18

**Authors:** Rajeev Bharadwaj, Deb Boruah, Bhupen Barman, Suman Kalita

**Affiliations:** 1 All India Institute of Medical Sciences, Guwahati, India

**Keywords:** hypertrophic cardiomyopathy, left ventricle outflow tract obstruction, mid-cavitary obstruction, heart failure, beta-blocker therapy, conservative management, risk stratification, hipertrofinė kardiomiopatija, kairiojo skilvelio obstrukcija, vidurinės ertmės obstrukcija, širdies nepakankamumas, beta adrenoblokatorių terapija, konservatyvus gydymas, rizikos stratifikacija

## Abstract

Hypertrophic cardiomyopathy (HCM) with mid-cavitary obstruction (MCO) is an uncommon condition affecting approximately 10% of HCM patients which is associated with serious outcomes, including sudden cardiac death and heart failure. We present the case of a 43-year-old male with type 2 diabetes mellitus who experienced worsening dyspnea and palpitations, leading to the diagnosis of HCM with MCO without outlet obstruction. Cardiac MRI with gadolinium contrast reveals significant mid-cavitary obstruction without left ventricular outflow tract obstruction with a scar burden of 27% and the absence of other high-risk factors such as apical aneurysm and NSVT runs on Holter monitoring. The patient was treated conservatively with the oral beta-blocker therapy, resulting in symptomatic improvement. Given the high risk of an adverse outcome, it is crucial to recognise MCO early and provide the appropriate treatment. This case report discusses the presentation, diagnosis, and management of a patient with HCM and mid-cavitary obstruction, highlighting the unique treatment considerations associated with this condition.

**Take home message:**

1. Mid-cavitary obstruction (MCO) in hypertrophic cardiomyopathy (HCM) is associated with high-risk outcomes of sudden cardiac death and heart failure.
2. While beta-blockers can improve symptoms in many MCO patients, treatment should be personalized based on the symptom severity and risk factors.
3. Patients with MCO are at risk of complications like apical aneurysms, thromboembolism, and arrhythmias.

## Abstract (it)

Hipertrofinė kardiomiopatija (HCM) su vidurinės ertmės obstrukcija (MCO) yra nedažna būklė, būna maždaug 10 proc. HCM sergančių pacientų ir susijusi su rimtais padariniais, įskaitant staigią širdies mirtį ir širdies nepakankamumą. Pateikiame 43 metų vyro, sergančio antro tipo cukriniu diabetu, atvejį, kai jam paūmėjo dusulys ir širdies plakimas, todėl buvo diagnozuota HCM su MCO obstrukcijos. Širdies MRT su gadolinio kontrastu atskleidė reikšmingą vidurinės ertmės obstrukciją be kairiojo skilvelio nutekamojo trakto obstrukcijos, randų našta sudarė 27 proc., o kitų didelės rizikos veiksnių, tokių kaip apikalinė aneurizma ir NSVT, Holterio stebėsenos metu nebuvo. Pacientas buvo gydomas konservatyviai – geriamaisiais beta adrenoblokatoriais, todėl simptomai pagerėjo. Atsižvelgiant į didelę neigiamos baigties riziką, labai svarbu anksti atpažinti MCO ir skirti tinkamą gydymą. Šiame atvejo aprašyme aptariamas paciento, sergančio HCM ir vidurinės ertmės obstrukcija, pristatymas, diagnozė ir gydymas, pabrėžiant unikalius su šia būkle susijusius gydymo aspektus.

## Introduction

*Hypertrophic Cardiomyopathy* (HCM) with *Mid-Cavitary Obstruction* (MCO) occurs in approximately 10% of HCM patients [1]. Impedance to flow at the middle of the left ventricle (LV), i.e., the so-called mid-cavitary obstruction (MCO), is a distinct and recognized phenotype of HCM, occurring as a result of segmental mid-septal hypertrophy and hypercontractility of the lateral ventricular wall along the hypertrophied papillary muscles [2].

In large cohorts of HCM, MCO has been associated with poor outcomes, including progression to end-stage heart failure, ventricular arrhythmia and sudden cardiac death [3]. Patients with MCO tend to have more symptoms compared to those without it [4]. This manuscript aims to present a rare case of *Mid-Cavitary Obstruction* (MCO) in *Hypertrophic Cardiomyopathy* (HCM) without *Left Ventricular Outflow Tract Obstruction* (LVOTO) and to highlight the role of advanced imaging in diagnosis, risk stratification, and the successful use of the conservative beta-blocker therapy. By sharing this case, we aim to contribute to the understanding of the challenges associated with MCO and its management, emphasizing the need for personalized treatment optimize patient outcomes.

## Case description

A 43-year-old male with type 2 diabetes mellitus presented with worsening dyspnoea for the previous 4 to 6 months (New York Heart Association Class II), and reported palpitation with exertion. He did not have any experience of syncope or angina, and there was no family history of sudden cardiac death in the previous three generations. His blood counts, renal function, and lipid profiles were normal. His NTproBNP levels were 2380 pg/ml. His 2D echocardiography revealed a posterior LV wall thickness of 16 mm and an interventricular septal thickness of 18 mm, a resting mid-cavitary gradient with pulse wave doppler of 36 mmHg, but no *Left Ventricular Outflow Obstruction* (LVOT) was noted. The left ventricular ejection fraction was 60%, and there were no valvular lesions. The left atrial size measured 38 mm, and Grade I left ventricular diastolic dysfunction was observed. The diagnosis of mid-ventricular obstruction was considered as the mid-ventricular gradient was more than 30mmHg [4].

**Figure 1:**
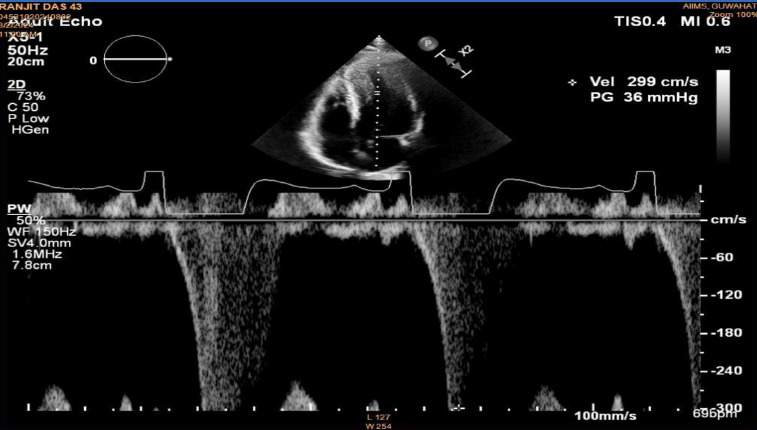
2 D Echocardiography with Pulse Doppler showing mid-cavitary gradient of 36 mm Hg (above 30 mm Hg is diagnostic cut-off for MCO).

**Figure 2:**
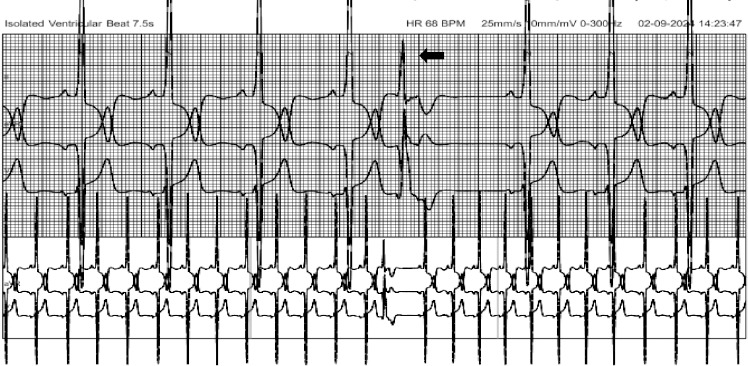
An excerpt from 24-hour Holter recording showing the sinus rhythm and a PVC complex (black arrow) with a compensatory pause. His 24-hour Holter showed no runs of NSVT and PVC burden of <1%.

**Figure 3:**
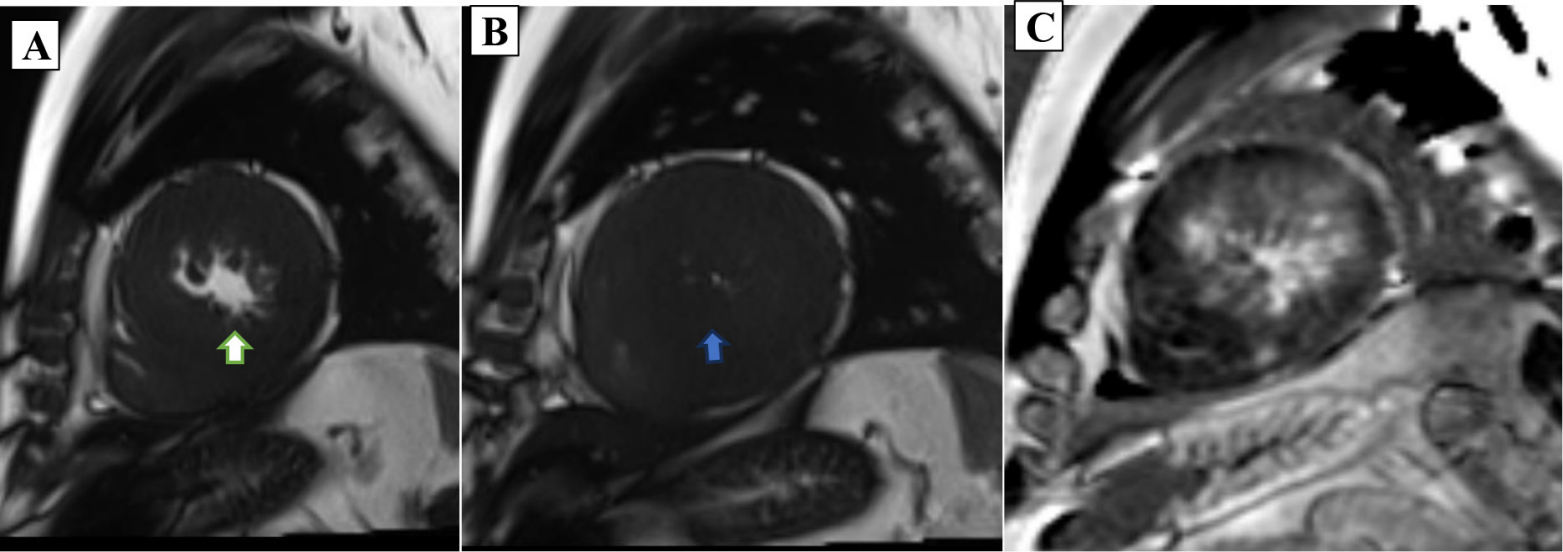
Left ventricular short axis *Cardiovascular Magnetic Resonance* (CMR) cine images in end-diastole with a white arrow (A) and end-systole with a blue arrow (B) showing near total LV cavity obliteration (MCO). (C) Identical imaging view in the same patient with gadolinium-diethylenetriaminepentaacetic acid showing extensive late gadolinium enhancement (Subendocardial to transmural).

**Figure 4:**
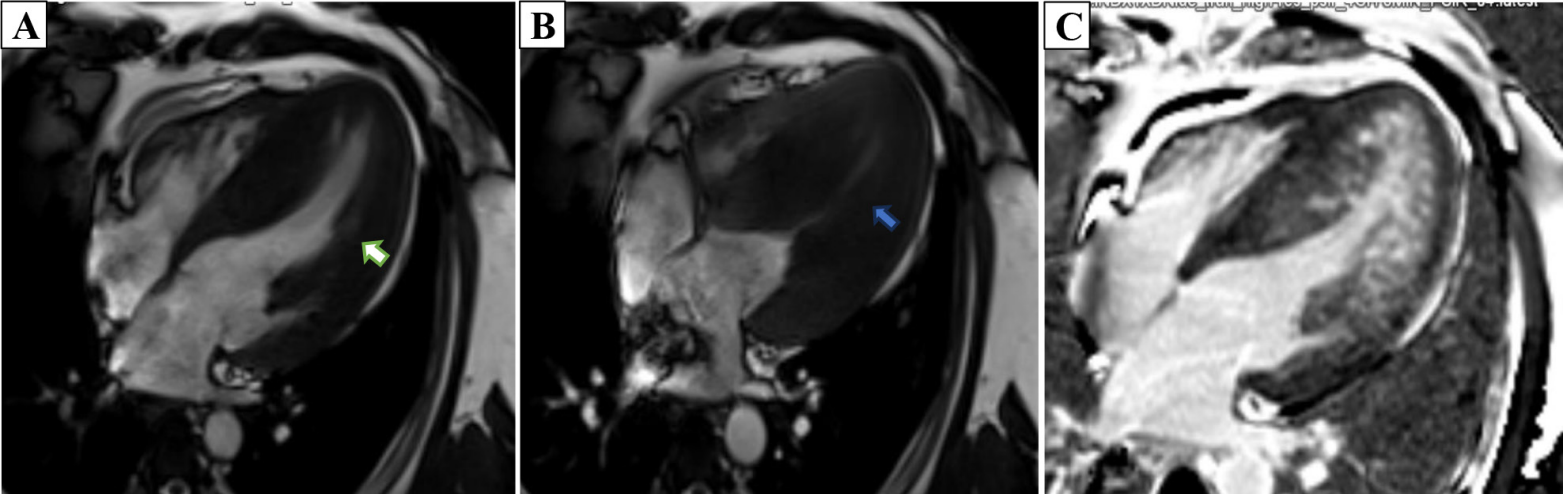
Left ventricular 4-chamber cardiovascular magnetic resonance (CMR) cine images in end-diastole (white arrow – A) and end-systole (blue arrow – B) showing LV MCO. (C) Identical imaging view in the same patient with gadolinium-diethylenetriaminepentaacetic acid showing extensive late gadolinium enhancement from mid ventricle to apex (Subendocardial to transmural).

Cardiac MRI was performed by using a *Magnetom Vida 3T* (*Siemens Healthcare*) apparatus, with postprocessing done via dedicated *Circle* (cvi42) software. The MRI revealed LV mid-cavitary obstruction on TRUE FISP cine sequences across long-axis, four-chamber, and short-axis planes, late gadolinium enhancements images, and a 4D flow study. Diffuse asymmetrical hypertrophy of the LV walls and IVS were noted with more involvement of the LV mid cavity along with hypertrophied papillary muscles. Mild to moderate narrowing of the mid cavity of the LV cavity noted with a pressure gradient of 43 mmHg across the obstruction on 4D flow Cardiac MRI(CMR). No LVOT obstruction or systolic anterior motion (SAM) were noted. Extensive transmural as well as subendocardial LGE enhancement were seen in the thickened LV walls and IVS. Marked enhancement was seen in the hypertrophied LV walls and noted in the LV mid cavity region. A scar burden of 27% was noted within the hypertrophied LV walls and IVS. Grade-II LV diastolic dysfunction was noted with aortic regurgitation with a grade of 12% with mild MR.

A 24-hour Holter monitoring by using the HSCRIBE Holter analysis system showed no runs of *Non-Sustained Ventricular Tachycardia* (NSVT), which is defined as ≥3 consecutive ventricular beats at a rate of ≥120 beats per minute and <30 seconds in duration and was otherwise unremarkable, aside from occasional ventricular ectopic beats. Risk stratification for sudden cardiac death over 5 years using the HCM risk score was 3.7 % [5,6]. In accordance with the ESC 2023 guideline on the management of HCM and considering the absence of additional risk factors, such as NSVT or a high burden (>10%) of *Premature Ventricular Contractions* (PVCs), the patient was treated conservatively with the oral beta-blocker therapy despite a high scar burden of 27% [5]. He was prescribed Metoprolol succinate extended-release at a dosage of 25 mg. Following the treatment, his symptoms improved, and he was advised a regular follow-up with 2D Echocardiography at 6-monthly intervals for further dose titration of beta blockers and close monitoring for complications. He was also advised genetic analysis for further risk assessment and prognostic information.

## Discussion

*Hypertrophic Cardiomyopathy* (HCM) with *Mid-Cavitary Obstruction* (MCO) is recognized as a distinct clinical condition which often leads to poor outcomes, although the optimal treatment strategies remain uncertain and are typically determined based on the severity of the obstruction [7]. Numerous case reports and cohort studies have been published, exploring medical treatment options and prognosis for patients with HCM complicated by MCO [1,8]. Despite these efforts, there remains a lack of consensus regarding the best approach for managing this subset of HCM patients, especially concerning the use of beta blockers.

Beta blockers are a cornerstone in the management of HCM with the *Left Ventricular Outflow Tract Obstruction* (LVOTO), where they help reduce the heart rate, improve myocardial relaxation, and alleviate symptoms such as dyspnoea and syncope. However, the role of beta blockers in managing MCO in HCM is not as clearly defined. A case report by Tezuka et al. demonstrated significant reduction in the mid ventricular gradient with the use of beta blockers. This decrease in the pressure gradient was accompanied by an improvement in the diastolic function at a three-month follow-up [9]. However, it is important to note that the response to such a therapy is not uniform across all patients. The efficacy of beta blockers in MCO can vary depending on the specific characteristics of the obstruction. Given the complex nature of MCO and the heterogeneity of patient responses, further studies are still necessary to fully understand the extent of the benefit that beta blockers may provide for this specific patient population. In addition, Tezuka et al. started bisoprolol treatment at a low dose (0.625 mg/day) with the dosage being gradually increased, while taking the fact that the patient was elderly into consideration. This highlights that the use of beta blockers is also individualized depending on the patient’s clinical scenario.

Beyond medical therapy, the management of HCM with MCO can be further complicated by the development of complications, such as apical aneurysms. Patients with MCO who develop apical aneurysms are at an elevated risk for adverse events, including sudden cardiac death (SCD), thrombus formation within the aneurysm, and progressive heart failure [10]. Approximately 20% of patients with MCO go on to develop apical aneurysms during long-term follow-up, according to several studies [1,11]. In this high-risk subgroup, the combination of beta blockers and anticoagulation therapy may serve as first-line treatments, aiming to reduce the risk of thromboembolism and improve the long-term outcomes [9].

Risk stratification is essential for the management and follow-up of patients with *Hypertrophic Cardiomyopathy* (HCM). For adult patients with HCM who present with at least one risk factor or are assessed as being at high risk (≥6%) using the *Sudden Cardiac Death* (SCD) risk prediction model, the implantation of an *Implantable Cardioverter Defibrillator* (ICD) should be considered for primary prevention [5]. The risk factors that contribute to this decision include age, the presence of NSVT, maximum left ventricular wall thickness, a family history of sudden cardiac death in young individuals, syncope, left atrial diameter, and LVOTO. In cases where these risk factors are absent, ICD implantation may still be considered for primary prevention under certain circumstances. For example, if NSVT is detected on ambulatory ECG, if extensive myocardial fibrosis is indicated on *Cardiac Magnetic Resonance* (CMR), or if a moderate risk (≥4%, <6%) is identified by using the SCD risk prediction model, ICD implantation could be appropriate [12]. Additionally, it has been noted that patients with mid-cavitary obstruction (MCO) have a higher incidence of syncope compared to those without MCO [13]. A study by Ryozo Maeda et al. found that the rate of ICD interventions in patients with MCO was 6.2% per year over an average follow-up period of 6.5 years. This is in contrast to a meta-analysis by Schinkel AFL et al., which reported an ICD intervention rate of 3.3% per year in a broader cohort of HCM patients [14,15]. These findings highlight the increased risk of adverse events, including the need for ICD interventions, in patients with MCO compared to HCM.

For patients with severe MCO and a pressure gradient exceeding 50 mmHg, along with symptoms of dyspnoea (NYHA class III or IV), surgical intervention may be required. Options include transapical or transaortic myectomy, or a combined approach. The decision between these surgical strategies hinges on the underlying anatomy and severity of the obstruction. It is critical to differentiate between MCO and LVOT obstruction, as standard septal reduction therapies (such as septal myectomy) may be insufficient for the management of MCO. For patients with MCO, isolated transapical or combined transapical/transaortic myectomy may offer more effective relief of the obstruction. The Mayo Clinic’s retrospective cohort study, which evaluated 196 patients undergoing septal myectomy for HCM with MCO, concluded that transapical myectomy is the most effective method to relieve MCO in these patients [16]. Similarly, Tang et al. suggested that transapical myectomy should be considered in patients with a long segment of MCO, those with limited exposure of the midventricular region, or patients who have a concomitant apical aneurysm [17].

In terms of treatment of refractory cases of HCM with MCO, distal ventricular pacing has been shown to reduce the obstruction and improve the symptoms [18].ṁ

## Conclusion

This case illustrates the clinical complexities of managing mid-cavitary obstruction in hypertrophic cardiomyopathy. Although MCO is linked to adverse outcomes, early identification and appropriate treatment can lead to significant symptom relief. Although, cardiac MRI revealed a scar burden exceeding 15%, yet due to the absence of other high-risk factors such as apical aneurysm and *Non-Sustained Ventricular Tachycardia* (NSVT) runs, along with significant symptomatic improvement with the beta-blocker therapy, the decision was made to continue regular follow-up with 6-monthly assessment with 2D Echocardiography rather than pursue surgical intervention. He was further advised follow up with the clinical exome sequencing for further risks stratification. The need for continuous monitoring and evaluation of treatment efficacy is paramount, particularly as patients with MCO may have unique complications such as apical aneurysm formation.

## Limitations

The major limitation is the lack of long-term follow-up data which limits the ability to comment on outcomes, reliance on a single case for representation and the absence of genetic analysis for risks stratification. A longer duration of Holter monitoring (>24 hours) on the follow-up is needed for the detection of NSVT runs. Future research should focus on delineating the long-term outcomes of patients with MCO and optimizing the therapeutic strategies, including potential surgical interventions, to enhance the quality of care for individuals affected by this challenging condition.
